# SelenoDB 2.0: annotation of selenoprotein genes in animals and their genetic diversity in humans

**DOI:** 10.1093/nar/gkt1045

**Published:** 2013-11-03

**Authors:** Frédéric Romagné, Didac Santesmasses, Louise White, Gaurab K. Sarangi, Marco Mariotti, Ron Hübler, Antje Weihmann, Genís Parra, Vadim N. Gladyshev, Roderic Guigó, Sergi Castellano

**Affiliations:** ^1^Department of Evolutionary Genetics, Max Planck Institute for Evolutionary Anthropology, Leipzig 04103, Germany, ^2^Bioinformatics and Genomics Programme, Centre for Genomic Regulation (CRG), Dr. Aiguader 88, 08003 Barcelona, Spain, ^3^Universitat Pompeu Fabra (UPF), 08003 Barcelona, Spain and ^4^Department of Medicine, Division of Genetics, Brigham and Women’s Hospital and Harvard Medical School, Boston, MA 02115, USA

## Abstract

SelenoDB (http://www.selenodb.org) aims to provide high-quality annotations of selenoprotein genes, proteins and SECIS elements. Selenoproteins are proteins that contain the amino acid selenocysteine (Sec) and the first release of the database included annotations for eight species. Since the release of SelenoDB 1.0 many new animal genomes have been sequenced. The annotations of selenoproteins in new genomes usually contain many errors in major databases. For this reason, we have now fully annotated selenoprotein genes in 58 animal genomes. We provide manually curated annotations for human selenoproteins, whereas we use an automatic annotation pipeline to annotate selenoprotein genes in other animal genomes. In addition, we annotate the homologous genes containing cysteine (Cys) instead of Sec. Finally, we have surveyed genetic variation in the annotated genes in humans. We use exon capture and resequencing approaches to identify single-nucleotide polymorphisms in more than 50 human populations around the world. We thus present a detailed view of the genetic divergence of Sec- and Cys-containing genes in animals and their diversity in humans. The addition of these datasets into the second release of the database provides a valuable resource for addressing medical and evolutionary questions in selenium biology.

## INTRODUCTION

Selenoproteins are proteins that contain the amino acid selenocysteine (Sec) as one of their constituent residues. Sec, the 21st amino acid in the genetic code, is analogous to the amino acid cysteine (Cys) in its molecular structure with an atom of selenium replacing that of sulfur in Cys. An in-frame UGA (stop) codon in conjugation of a SElenoCysteine Insertion Sequence (SECIS) element, an RNA secondary structure in the mRNA of selenoproteins, codes for a Sec residue instead of terminating protein synthesis (1).

The discovery of Sec itself and the associated translation mechanism are relatively recent (2–5). The dual and seemingly ambiguous nature of the UGA codons does not make it any easier to identify and annotate selenoprotein genes using standard gene annotation pipelines. This has lead to many annotation errors in the past, because most gene annotations pipelines still solely rely on using UGA codons to determine the end of open reading frames (ORFs), which in the case of Sec will be completely wrong.

The errors in the annotation of selenoproteins in sequenced genomes were our primary motivation behind developing SelenoDB. With SelenoDB 1.0 (6) as the first step in this direction, we correctly annotated selenoprotein genes in a small number of species. This release of the database has contributed to the study of Sec and selenoproteins in the last few years (7–12). Since the release of the first version of SelenoDB, the genomes of many more animal species have been sequenced. Unfortunately, the lack of correct annotations of selenoproteins persists today for the majority of these species. For example, Ensembl (13) now provides gene annotations for dozens of animal species but, with the exception of the human genome, the annotation of selenoproteins in these species contains many errors (e.g. truncated gene structures stopping at or skipping the Sec residue). In SelenoDB 2.0, we provide the correct gene annotations for selenoproteins in 58 of these species, including humans. Thus, we provide a resource to further study the biology of selenium-containing proteins across metazoans.

Selenium requirement in humans may be influenced by genetic variation in selenoprotein genes (14). A number of single-nucleotide polymorphisms (SNPs) in different selenoprotein genes have been shown to have functional consequences and may affect the efficacy of selenium utilization (15–20). To put this research in the context of selenoprotein genetic diversity in humans, it is necessary to obtain an unbiased catalog of the genetic variants and their frequencies in human populations. With SelenoDB 2.0, we present such catalog from a large resequencing study of human populations across the world. Both medical and evolutionary studies benefit from these data.

## A SUMMARY OF SelenoDB 1.0

We released version 1.0 of SelenoDB in 2008 with an initial set of genomic annotations. In this release, we put special emphasis on the correct annotation of the human selenoproteome. Gene prediction was performed using either genewise (21), exonerate (22) or spidey (23). SECIS predictions were obtained using the SECISearch program, release 2.19 (24). We manually curated all genes and SECIS predictions.

The database could be searched using a number of ways, from simple keyword searches to more flexible and powerful advanced searches by grouping features together. Moreover, with a fair amount of familiarity with SQL and the database schema, users could dig much deeper into the database using command-line queries. The query results are displayed in the feature reports for genes, transcripts, proteins or SECIS elements in one or more species. These reports include information about gene and protein names, family and subfamily names, species and its taxonomical classification and the genomic or protein annotation itself. Even though this first release of SelenoDB had few annotations, it allowed us to develop a robust relational database implemented in MySQL 5.0. The database schema was designed to store non-standard genes with recoded codons, alternative translation initiation and termination sites, RNA secondary structures and other unusual features. We take advantage of the versatility of this framework in the design of SelenoDB 2.0.

## WHAT IS NEW IN SelenoDB 2.0?

The structure and interface of the database in SelenoDB 2.0 retains most of the features of release 1.0 with a number of enhancements. In particular, the second release of SelenoDB is now able to accommodate the annotation of multiple transcripts per gene. We provide them for humans only. In addition, in order to cope with the growing number of sequenced genomes, we have now switched to fully automatic annotations using Selenoprofiles (25), a homology-based annotation pipeline for selenoprotein genes. This has allowed us to obtain selenoprotein gene annotations for more than 50 new genomes. In addition, we have now included high-quality SNP data for a worldwide sample of humans. Table 1 shows a comparison of the features present in the first and second release of SelenoDB.

**Table 1.** Comparison of features in the first and second releases of SelenoDB

Features	Release 1.0	Release 2.0
Number of species	8	59
Number of protein families	20	28
Number of genes	81	2801
Alternative transcripts	Not present	For one species
Variation data	Not present	For one species
Curation method	Manual	Manual and automatic

## GENE ANNOTATION

### Manual annotation of human selenoprotein genes

**Figure 1.**
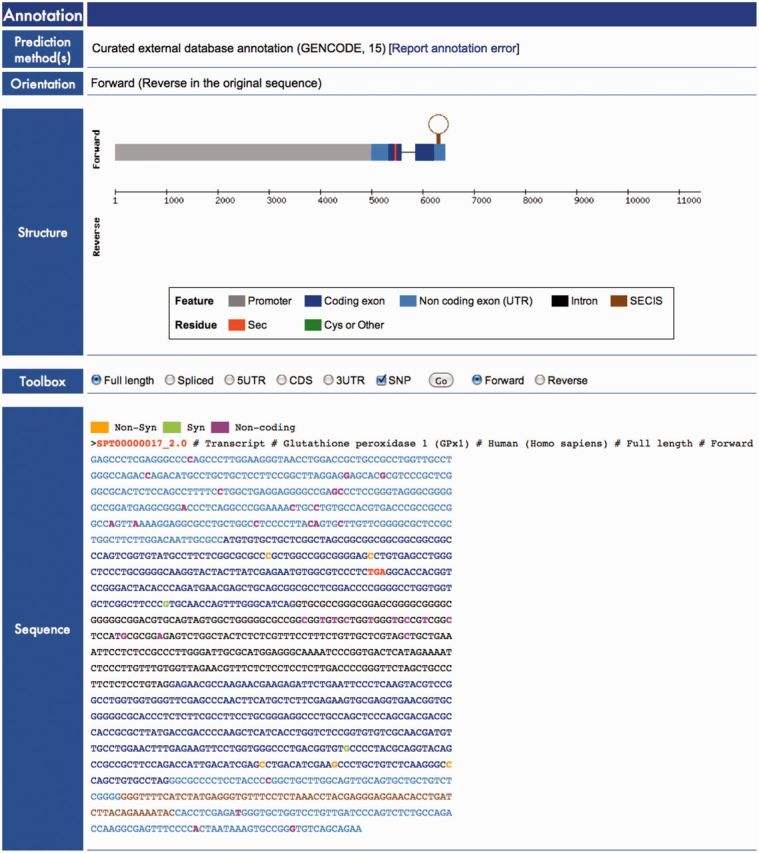
Human *glutathione peroxidase 1* (*GPx1*) transcript. Note the non-synonymous, synonymous and non-coding SNPs annotated in the transcript sequence. The gene structure and transcript sequence is shown in forward despite being annotated in the reverse strand of the reference human genome.

SelenoDB 2.0 includes a manually curated annotation of human selenoproteins, Cys-containing homologs and genes involved in the metabolism of selenium and Sec derived from the GENCODE annotation (release 15), which we contributed to produce (26). Thus, we incorporate this annotation into the new release of SelenoDB (Figure 1), including a number of alternative splice variants. For each gene, however, only those transcripts that are classified as protein coding (containing an ORF) are included.

### Automatic annotation of non-human selenoprotein genes

Using Selenoprofiles (25), we present a comprehensive annotation of selenoprotein genes, Cys-containing homologs and genes involved in the metabolism of selenium and Sec in a large number of Metazoan genomes from Ensembl (release 68). This set of 57 animal species contains representatives of several taxonomic groups: Mammalia (38), Actinopterygii (7), Aves (3), Testudines (1), Squamata (1), Amphibia (1), Coelacanthimorpha (1), lampreys (1) and the non-vertebrate Tunicata (2), Insecta (1) and Nematoda (1). In addition, we annotate the *Saccharo-myces cerevisiae* genome. This yeast genome lacks selenoproteins but contains selenoprotein homologs with Cys in the place of Sec.

**Figure 2.**
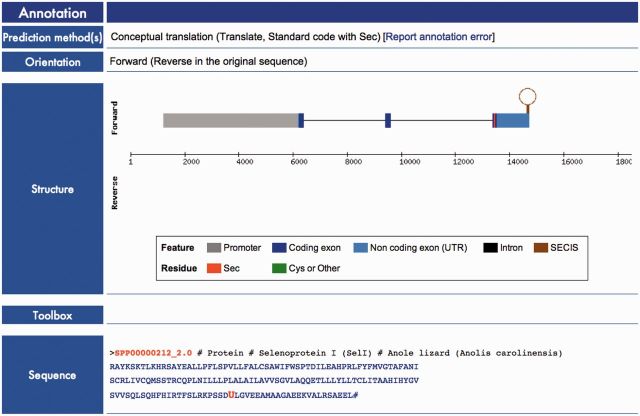
Lizard *selenoprotein I* (*SelI*). Note the predicted Sec (U) in the protein sequence as well as the TAA (#) termination codon. The N-terminal of the protein is missing due to lack of sequence similarity between the protein sequence profile used by Selenoprofiles and a divergent lizard genome sequence.

Selenoprofiles is a homology-based annotation pipeline, specially designed for the detection of selenoprotein genes in target genome sequences. It produces accurate gene predictions using a set of manually curated profiles, one for each known protein family. Each profile is built from a multiple amino acid sequence alignment of representative members of the family, including the Sec residue. Unlike other gene prediction pipelines, Selenoprofiles is able to correctly predict selenoprotein genes. The genome sequence is scanned using the psi-blast program (27) with a position-specific scoring matrix derived from the profile. Selenoprofiles predicts the exonic structures of the candidate genes using the splice alignment programs exonerate (22) and genewise (21), while maintaining the Sec residue in the gene structure predictions. The predictions by the various programs are then merged, processed and finally filtered, using filters tuned for each protein family. The usually lower levels of similarity of the C- and N-terminal between orthologous proteins can result in Selenoprofiles predicting only the central part of selenoproteins in divergent animal genomes (Figure 2).

### New protein families

We have annotated 23 selenoprotein families (28) in SelenoDB 2.0 (Table 2). Of these, SelJ (29), Fep15 (30), SelL (31) and FrnE (DsbA) (32) are new to the database. Although Fep15 is distantly related to the Sel15 family, we annotate them as two distinct protein families. On the other hand, the two families SelV and SelW share a high sequence similarity (although SelV has an additional N-terminal domain) (33), and are merged into a single SelW family by the Selenoprofiles pipeline. As a result, non-human species have annotations for the SelW family only.

**Table 2.** Protein families annotated in the second release of SelenoDB

**Selenoprotein families (****28****)**	
Glutathione peroxidase (GPx)	
Iodothyronine deiodinadse (DI)	
15 kDa selenoprotein (Sel15)	
15 kDa selenoprotein-like protein (Fep15)	
FrnE (FrnE)	
Methionine sulfoxide reductase A (MsrA)	
Selenophosphate synthetase (SPS)	
Selenoprotein H (SelH)	
Selenoprotein I (SelI)	
Selenoprotein J (SelJ)	
Selenoprotein K (SelK)	
Selenoprotein L (SelL)	
Selenoprotein M (SelM)	
Selenoprotein N (SelN)	
Selenoprotein O (SelO)	
Selenoprotein P (SelP)	
Selenoprotein R (SelR)	
Selenoprotein S (SelS)	
Selenoprotein T (SelT)	
Selenoprotein U (SelU)	
Selenoprotein V (SelV)	
Selenoprotein W (SelW)	
Thioredoxin reductase (TR)	
**Sec insertion machinery families**	
Eukaryotic elongation factor (eEFSec)	
Phosphoseryl-tRNA kinase (PSTK)	
SECIS binding protein 2 (SBP2)	
Selenocysteine synthase (SecS)	
tRNA Sec 1 associated protein 1 (SECp43)	

tRNA, transfer RNA.

In addition, we have annotated the structure of five additional gene families associated with the Sec insertion machinery (34) (Table 2). The *O-phosphoseryl-tRNA^Sec^* (*PSTK*), *Selenocysteine synthase* (*SecS*) and the associated protein 43 (*SECp43*) genes are annotated for the first time in SelenoDB.

### Orthology assignment

Selenoprofiles identifies the family (e.g*.* glutathione peroxidase) but not the subfamily (e.g. glutathione peroxidase 1) of a predicted protein because this entails phylogenetic analysis with a species of reference. That is, a species where all the members of a protein family are reliably assigned to subfamilies. This is the case only for the selenoprotein families annotated in the human genome (6). Therefore, for each family in the non-human species we infer a phylogenetic tree that includes the homologous protein family in humans using the PhylomeDB pipeline (35). In such trees, we distinguish between duplication and speciation nodes and use the latter to identify orthologous genes between the non-human species and human (36). We assign the subfamily of the human selenoproteins to their non-human orthologs.

In some cases, the orthology relationship between proteins is not one to one (e.g. in the case of a duplication event in a non-human protein family). In such cases, we chose not to assign a subfamily based on the reference human proteins.

### SECIS annotation

The SECIS element is a RNA stem–loop found in the selenoprotein mRNAs, essential for Sec insertion. In eukaryotes, it resides in the 3′-UTR (untranslated region) and can be classified in two classes, type I and type II with the latter possessing an additional helix and a short apical loop. The structure adopts a kink-turn motif through the non-canonical base pairs AG-GA in the quartet, the most conserved region in eukaryotic SECIS elements (37). Computational identification of SECIS elements has been used in the past to identify selenoprotein genes (24,38). Recently, the SECISearch method has been improved. SECISearch3 (39) is a pipeline for the identification of eukaryotic SECIS elements that combines several methods for RNA structure prediction. A filter removes unlikely SECIS candidates, checking their structural features and thermodynamic stability. SelenoDB 2.0 includes SECIS elements predicted by SECISearch3 in the 6-kb region downstream from the coding sequence of all predicted selenoprotein genes (Figures 1 and 2). The end of the predicted coding region by Selenoprofiles is then extended up to the predicted SECIS to be annotated as the 3′-UTR of the gene.

## VARIATION DATA

SelenoDB 2.0 includes intra-specific diversity data for the first time and, in doing so, gives what is currently the best view of human variation in selenoprotein genes, Cys-containing homologs and genes involved in the Sec insertion machinery. We include SNP data from 928 human samples from the CEPH HGDP panel (40). These samples are from 53 populations spanning a diversity of geographic locations from Africa, Middle East, Europe, Asia, Oceania and America. All samples were sequenced on the same platform and the SNPs were stringently filtered to ensure high quality and reliability.

### Exome capture and sequencing

To obtain the human data, we used an Agilent custom array (Agilent Technologies) to target all exons plus 200 bp of the surrounding introns and 2000 bp upstream (to include promoter regions) of genes in Table 2. Target capture was performed in batches of pooled libraries with around 90 samples per pool. Libraries were sequenced using the Illumina GAIIx platform yielding 76 bp paired-end reads. Base calling was performed with Ibis (41).

### SNP calling

Human sequences were mapped to the human reference genome (hg19) using BWA (42) yielding an average on-target coverage of 20x and 18x per individual and per gene. Sequences with a mapping quality <25 were filtered out and GATK IndelRealigner (43,44) was used to improve sequence alignment in indel regions. A set of secondary target regions was defined for SNP calling. These were defined as the whole gene including all exons, introns and UTRs plus 2500 bp upstream and downstream of the longest transcript in each gene. SNPs and indels were called separately in the secondary target regions, using GATK UnifiedGenotyper version 2.2 (44).

The initial GATK output was put through a comprehensive set of filters to remove sites that: (i) had a coverage below 8x in more than 50% of samples; (ii) had an average coverage above 100x; (iii) were indels or SNPs within 5 bp of an indel; (iv) were trialellic sites; (v) had a GATK SNP quality <20 and (vi) a strand bias (SB) >10. We additionally filtered out human SNPs that did not have one-to-one human to chimpanzee correspondence in the Ensembl EPO 6 primate alignments (45,46) or were at sites identified as being prone to systematic error. This resulted in 4808 SNPs in the human samples for genes in Table 2.

## NEW INTERFACE FEATURES

**Figure 3.**
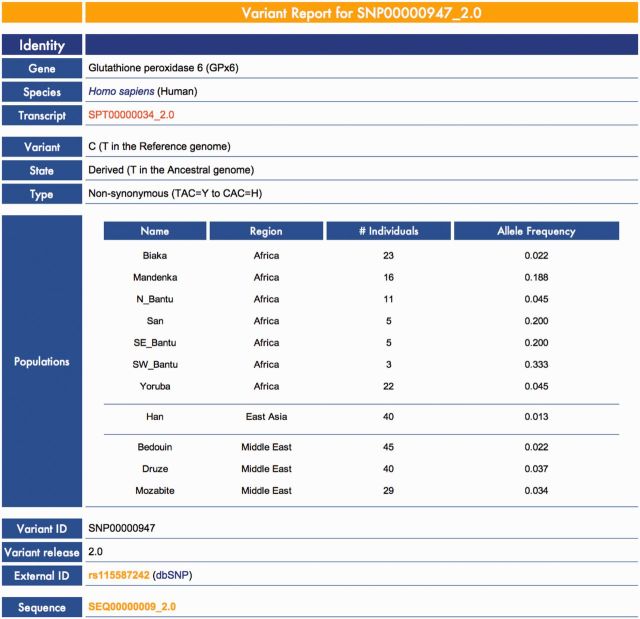
Variant report for a non-synonymous (Y to H) SNP in the human *GPx6* gene. An ancestral T (present in the genome of the ancestor of humans and chimpanzees) has mutated to C in humans reaching higher frequencies in some African populations. Populations are grouped according to their geographical region of origin.

The majority of search, display and sequence manipulation features found in SelenoDB 1.0 (6) remain in the second version of the database presented here. The annotation of alternative transcripts in the human genome and the inclusion of SNP data from humans are, however, responsible for a number of interface changes. First, the Annotation section of the Gene reports has been modified in order to display, when necessary, more than one transcript per gene. For each gene, a list of links to the transcript(s), promoter(s), protein(s) and SECIS(es) report(s) is now available. Second, within the ‘Sequence’ section of each transcript and protein report, the SNPs identified in our survey are displayed (Figures 1 and 2). A click on a SNP leads to the corresponding variant report (Figure 3), which includes the type (non-coding, synonymous or non-synonymous when coding), state (ancestral or derived with respect to the human–chimpanzee ancestor) and population frequencies of the SNP. In addition, SNPs for each species and/or genes can be obtained using the advanced search form.

## FUTURE DIRECTIONS

With the release of SelenoDB 2.0, we have provided a comprehensive annotation of selenoprotein genes across animal genomes. Two features provided for human selenoproteins in this release are the annotation of alternative transcripts and a worldwide catalog of genetic variation. It would be of interest to selenium researchers to have the annotation of alternative transcripts in other species as well as a sample of the genetic diversity of selenoproteins in non-human species.

## FUNDING

The Max Planck Society; the Plan Nacional and the Instituto Nacional de BIoinformatica (Spain) (to R.G.); National Institutes of Health grants (to V.N.G.) Funding for open access charge: Max Planck Society.

*Conflict of interest statement*. None declared.
